# High-Pressure Synthesis and Characterization of New Actinide Borates, *An*B_4_O_8_ (*An*=Th, U)

**DOI:** 10.1002/chem.201302378

**Published:** 2013-10-10

**Authors:** Ernst Hinteregger, Thomas S Hofer, Gunter Heymann, Lukas Perfler, Florian Kraus, Hubert Huppertz

**Affiliations:** [a] Institute of General, Inorganic and Theoretical Chemistry, Leopold-Franzens-Universität Innsbruck Innrain 80–82, 6020 Innsbruck (Austria), Fax: (+43) 512-507-57099; [b] Institute of Mineralogy and Petrography, Leopold-Franzens-Universität Innsbruck Innrain 52 f, 6020 Innsbruck (Austria); [c] AG Fluorchemie, Department Chemie, Technische Universität München Lichtenbergstrasse 4, 85747 Garching (Germany)

**Keywords:** actinides, borates, density functional theory, high-pressure chemistry, Raman spectroscopy

## Abstract

New actinide borates ThB_4_O_8_ and UB_4_O_8_ were synthesized under high-pressure, high-temperature conditions (5.5 GPa/1100 °C for thorium borate, 10.5 GPa/1100 °C for the isotypic uranium borate) in a Walker-type multianvil apparatus from their corresponding actinide oxide and boron oxide. The crystal structure was determined on basis of single-crystal X-ray diffraction data that were collected at room temperature. Both compounds crystallized in the monoclinic space group *C*2/*c* (*Z*=4). Lattice parameters for ThB_4_O_8_: *a*=1611.3(3), *b*=419.86(8), *c*=730.6(2) pm; *β*=114.70(3)°; *V*=449.0(2) Å^3^; *R*_1_=0.0255, *wR*_2_=0.0653 (all data). Lattice parameters for UB_4_O_8_: *a*=1589.7(3), *b*=422.14(8), *c*=723.4(2) pm; *β*=114.13(3)°; *V*=443.1(2) Å^3^; *R*_1_=0.0227, *wR*_2_=0.0372 (all data). The new *An*B_4_O_8_ *(An=*Th, U) structure type is constructed from corner-sharing BO_4_ tetrahedra, which form layers in the *bc* plane. One of the four independent oxygen atoms is threefold-coordinated. The actinide cations are located between the boron–oxygen layers. In addition to Raman spectroscopic investigations, DFT calculations were performed to support the assignment of the vibrational bands.

## Introduction

Over the last decade, our research into the high-pressure chemistry of borates has led to the synthesis of several new compounds with fascinating structures, owing to the efficient use of the multianvil technique.^1^ For example, we discovered the rare-earth borate Dy_4_B_6_O_15_,^2^ which was the first borate that exhibited edge-sharing BO_4_ tetrahedra. Later on, HP-NiB_2_O_4_ was synthesized,^3^ which was the first borate in which all of the [BO_4_]^5−^ tetrahedra showed a linkage through a common edge to a second tetrahedron, as well as HP-KB_3_O_5_,^4^ which simultaneously contained all three possible conjunction modes, that is, corner-sharing BO_3_ groups, corner-sharing BO_4_ units, and edge-sharing BO_4_ tetrahedra.

Following our interest in the high-pressure chemistry of alkali, alkaline-earth, transition-metal, and rare-earth borates, we decided to broaden our research activities into the field of actinide borates. This field of structural chemistry is highly topical, as reflected by the considerable number of new actinide borates with interesting structures and properties that have been synthesized within the last few years.^5^–^11^ A closer look at the existing compounds with *An*-B-O ternary systems only showed a few phases. Just six compounds with the actinide cations thorium, uranium, and americium are known, namely: Th(B_2_O_5_),^12^ ThB_66.8_O_0.36_,^13^ U(BO_3_)_2_,^14^ (UO_2_)(B_2_O_4_),^12^ AmB_9_O_18_,^11^ and AmBO_3_.^15^ To the best of our knowledge, no ternary actinide borates have been synthesized under high-pressure conditions so far. However, recent studies on the chemistry of high-pressure alkaline uranyl borates demonstrated the feasibility of this approach.^10^ Historically, Berzelius has already reported the possible presence of a thorium borate in a mineral that was found in Norway in 1824.^16^ Furthermore, there are several existing hydrated actinide borates of the actinides thorium, uranium, neptunium, plutonium, and americium. Research into actinide borates is of urgent importance in the question of the storage of nuclear waste. Owing to the high stability and insolubility of borates, they are of interest for the immobilization of nuclear waste. In this context, borates in which the metal cation is in the oxidation state 4+ have a special position, because the cation (especially cerium) can be regarded as a “dummy” for plutonium, owing to their comparable ionic radii. Herein, we describe the syntheses, single-crystal structural determinations, and Raman spectroscopic investigations of *An*B_4_O_8_ *(An=*Th, U), as well as quantum-chemical calculations of the harmonic vibrational frequencies of ThB_4_O_8_.

## Results and Discussion

**Figure 1:**
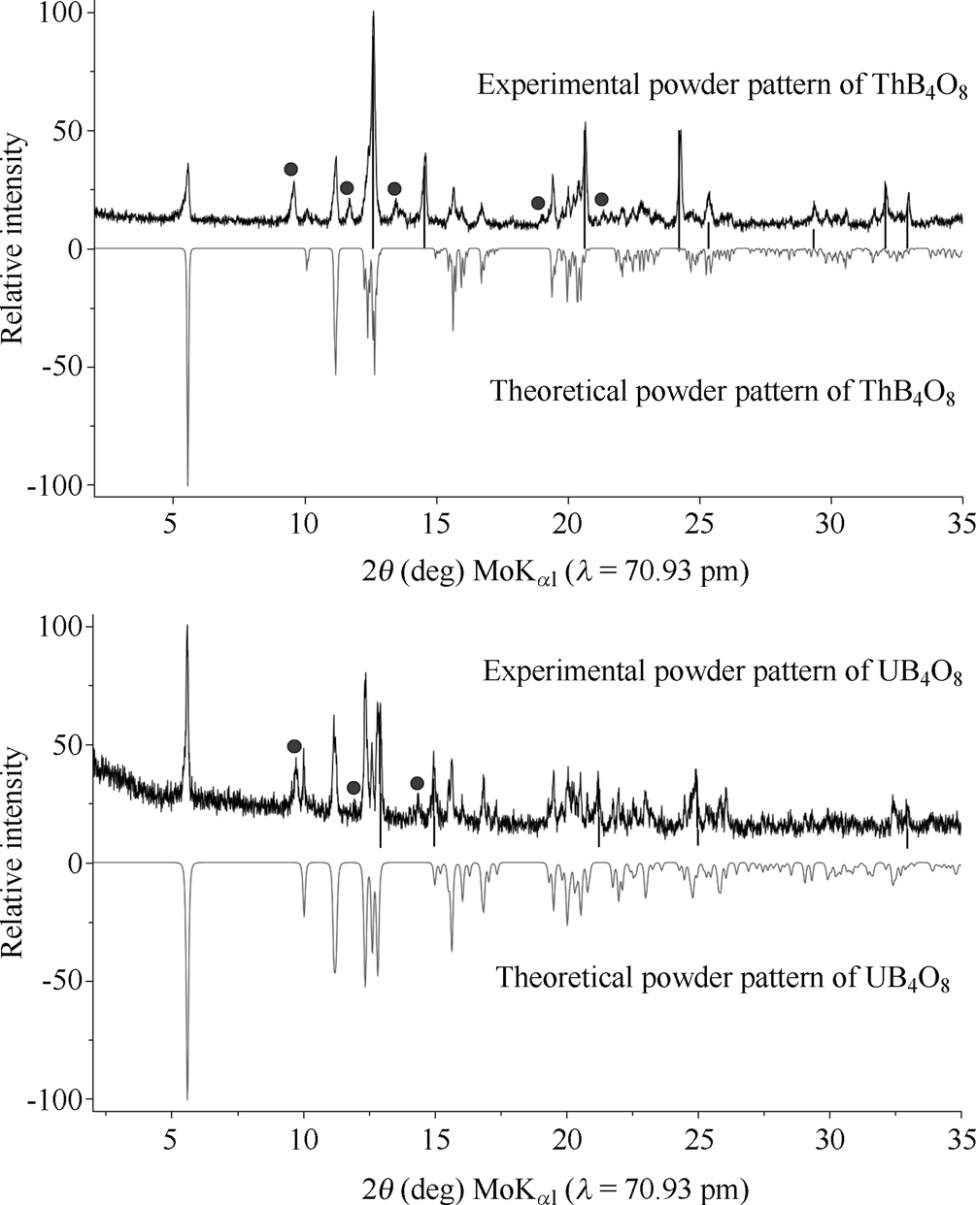
Top: Experimental and theoretical powder X-ray diffraction (PXRD) patterns of ThB_4_O_8_ (space group *C*2/*c*); the reflections of an unknown phase are indicated by circles. The reflections of ThO_2_ are marked with lines. Bottom: Experimental and theoretical PXRD patterns of UB_4_O_8_ (space group *C*2/*c*); reflections of an unknown phase are indicated by circles. The reflexions of UO_2.12_ are marked with lines. The theoretical PXRD patterns are based on the single-crystal diffraction data.

**Synthesis and crystal-structure analysis**: The compounds ThB_4_O_8_ and UB_4_O_8_ were synthesized from their corresponding actinide oxides and B_2_O_3_ under high-pressure, high-temperature conditions (5.5 GPa and 1100 °C for ThB_4_O_8_; 10.5 GPa and 1100 °C for UB_4_O_8_) in a 1000 ton multianvil press that was fitted with a Walker-type module. A detailed description of the syntheses is provided in the Experimental Section. Figure 1 shows the diffraction patterns of ThB_4_O_8_ (top) and UB_4_O_8_ (bottom), as well as reflections of the corresponding actinide oxide (marked with lines) and reflections of another still-unknown side product (marked with circles). The single-crystal intensity data were collected at room temperature on a Nonius Kappa-CCD diffractometer with graphite-monochromated Mo_Kα_ radiation (*λ*=71.073 pm). Tables 1, Table 2, and Table 3 list the details of the data collection and evaluation, as well as the positional parameters of the refinement. Interatomic distances and interatomic angles are listed in Table 4 and Table 5.

**Table 1:** Crystal data and structure refinement of *An*B_4_O_8_ (*An=*Th, U; space group *C*2/*c*); standard deviations are given in parentheses

Empirical formula	ThB_4_O_8_	UB_4_O_8_
molar mass [g mol^−1^]	403.28	409.27
crystal system	monoclinic	monoclinic
space group	*C*2/*c* (No. 15)	*C*2/*c* (No. 15)

Single-crystal data		
single-crystal diffractometer	Enraf–Nonius Kappa CCD	Enraf–Nonius Kappa CCD
radiation	Mo_Kα_ (*λ*=71.073 pm)	Mo_Kα_ (*λ*=71.073 pm)
*a* [pm]	1611.3(3)	1589.7(3)
*b* [pm]	419.86(8)	422.14(8)
*c* [pm]	730.6(2)	723.4(2)
*β* [°]	114.70(3)	114.13(3)
*V* [Å^3^]	449.0(2)	443.1(2)
formula units per cell	4	4
*ρ*_calcd_ [g cm^−3^]	5.97	6.14
crystal size [mm^3^]	0.08×0.15×0.05	0.02×0.02×0.01
*T* [K]	293(2)	293(2)
*µ* [mm^−1^]	33.22	36.64
*F*(000)	696	704
*θ* range [°]	2.8–30.0	2.8–30.0
range in *hkl*	±22, ±5, ±10	±22, ±5, ±10
total no. of reflections	2400	2382
independent reflections	655 (*R*_int_=0.0495)	640 (*R*_int_=0.0561)
reflections with [*I*≥2*σ*(*I*)]	653 (R*_σ_*=0.0345)	602 (R*_σ_*=0.0387)
data/parameters	655/61	640/61
absorption correction	multi-scan (Scalepack^[46]^)	multi-scan (Scalepack^[46]^)
GOF on *F*_i_^2^	1.175	1.059
final *R* indices [*I*≥2*σ*(*I*)]	*R*_1_=0.0254	*R*_1_=0.0187
	*wR*_2_=0.0652	*wR*_2_=0.0365
*R* indices (all data)	*R*_1_=0.0255	*R*_1_=0.0227
	*wR*_2_=0.0653	*wR*_2_=0.0372
largest diff. peak/hole [e Å^−3^]	5.925/−1.990	1.512/−1.191

**Table 2:** Atomic coordinates, Wyckoff positions, and equivalent isotropic displacement parameters (*U*_eq_ [Å^2^]) of *An*B_4_O_8_ (*An=*Th, U; space group *C*2/*c*); standard deviations are given in parentheses^[a]^

Atom	Wyckoff position	*x*	*y*	*z*	*U*_eq_
Th1	4*e*	0	0.19317(4)	0.25	0.0035(2)
O1	8*f*	0.2163(3)	0.146(2)	0.1675(7)	0.0033(7)
O2	8*f*	0.3428(3)	0.152(2)	0.4908(7)	0.0042(8)
O3	8*f*	0.3678(4)	0.3026(7)	0.2043(8)	0.0044(9)
O4	8*f*	0.4480(3)	0.261 (2)	0.0129(7)	0.0057(8)
B1	8*f*	0.3038(5)	0.304(2)	0.296(2)	0.003(2)
B2	8*f*	0.3584(6)	0.189(2)	0.008(2)	0.004(2)
U1	4*e*	0	0.16767(6)	0.25	0.0048(2)
O1	8*f*	0.2163(2)	0.1454(8)	0.1679(5)	0.0041(6)
O2	8*f*	0.3452(2)	0.1510(9)	0.4929(5)	0.0050(6)
O3	8*f*	0.3688(2)	0.3048(8)	0.2012(5)	0.0033(6)
O4	8*f*	0.4484(2)	0.2652(8)	0.0038(6)	0.0066(7)
B1	8*f*	0.3049(4)	0.304(2)	0.2961(8)	0.004(2)
B2	8*f*	0.3603(4)	0.187(2)	0.0062(8)	0.006(2)

[a] *U*_eq_ is defined as one third of the trace of the orthogonalized *U_ij_* tensor.

**Table 3:** Anisotropic displacement parameters (*U_ij_* [Å^2^]) for *An*B_4_O_8_ (*An=*Th, U; space group: *C*2/*c*); standard deviations are given in parentheses

Atom	*U*_11_	*U*_22_	*U*_33_	*U*_12_	*U*_13_	*U*_23_
Th1	0.0032(2)	0.0050(2)	0.0028(2)	0	0.0018(2)	0
O1	0.002(2)	0.003(2)	0.004(2)	0.000(2)	0.000(2)	0.001(2)
O2	0.007(2)	0.003(2)	0.003(2)	−0.001(2)	0.002(2)	0.001(2)
O3	0.002(2)	0.008(2)	0.005(2)	−0.001(2)	0.003(2)	0.000(2)
O4	0.008(2)	0.006(2)	0.005(2)	0.000(2)	0.004(2)	0.000(2)
B1	0.002(3)	0.005(3)	0.001(3)	−0.001(2)	−0.001(3)	0.001(2)
B2	0.004(3)	0.004(3)	0.003(3)	0.001(2)	0.002(3)	0.000(2)
U1	0.0044(2)	0.0053(2)	0.0047(2)	0	0.0021(2)	0
O1	0.003(2)	0.006(2)	0.002(2)	0.000(2)	−0.001(2)	0.000 (2)
O2	0.007(2)	0.002(2)	0.006(2)	0.001(2)	0.002(2)	0.001(2)
O3	0.004(2)	0.004(2)	0.003(2)	0.000(2)	0.002(2)	−0.001(2)
O4	0.005(2)	0.006(2)	0.012(2)	−0.002(2)	0.006(2)	−0.002(2)
B1	0.003(2)	0.006(2)	0.003(2)	−0.001(2)	0.000(2)	−0.001(2)
B2	0.009(3)	0.005(2)	0.005(2)	−0.002(2)	0.005(2)	0.002(2)

**Table 4:** Interatomic distances [pm] in *An*B_4_O_8_ (*An=*Th, U; space group *C*2/*c*), as calculated from the single-crystal lattice parameters; standard deviations are given in parentheses

ThB_4_O_8_		UB_4_O_8_	
Th1–O4	240.3(5) (×2)	U1–O4	231.4(4) (×2)
Th1–O4′	240.4(5) (×2)	U1–O4′	235.3(4) (×2)
Th1–O2	253.3(5) (×2)	U1–O3	249.4(5) (×2)
Th1–O3	259.7(5) (×2)	U1–O2	252.2(4) (×2)
Th1–O4′′	285.9(5) (×2)	U1–O4′′	300.2(4) (×2)
Ø=255.9		Ø=253.7	
B1–O2	144.3(8)	B1–O3	144.0(6)
B1–O3	144.3(9)	B1–O2	145.1(6)
B1–O1	148.4(8)	B1–O1	149.1(8)
B1–O1′	152.1(6)	B1–O1′	152.8(6)
Ø=147.3		Ø=147.8	
B2–O2	145.0(5)	B2–O2	144.5(6)
B2–O3	146.0(9)	B2–O4	144.6(7)
B2–O4	146.0(9)	B2–O3	144.8(6)
B–O1	150.9(8)	B2–O1	152.1(7)
Ø=147.0		Ø=146.5	

**Table 5:** Interatomic angles [°] in *An*B_4_O_8_ (*An=*Th, U; space group *C*2/*c*), as calculated from the single-crystal lattice parameters; standard deviations are given in parentheses

ThB_4_O_8_			
O3-B1-O2	110.8(6)	O2-B2-O4	109.9(5)
O3-B1-O1	112.3(5)	O2-B2-O3	110.4(5)
O2-B1-O1	109.5(4)	O4-B2-O3	102.2(5)
O3-B1-O1′	109.3(4)	O2-B2-O1	109.5(5)
O2-B1-O1′	106.3(5)	O4-B2-O1	110.9(5)
O1-B1-O1′	108.5(5)	O3-B2-O1	113.7(5)
Ø=109.5		Ø=109.4	

UB_4_O_8_			
O3-B1-O2	110.7(4)	O2-B2-O4	111.0(4)
O3-B1-O1	112.1(4)	O2-B2-O3	110.5(4)
O2-B1-O1	109.7(4)	O4-B2-O3	103.2(4)
O3-B1-O1′	109.0(4)	O2-B2-O1	110.4(4)
O2-B1-O1′	107.0(4)	O4-B2-O1	109.6(4)
O1-B1-O1′	108.1(4)	O3-B2-O1	111.9(4)
Ø=109.4		Ø=109.4	

**Figure 2:**
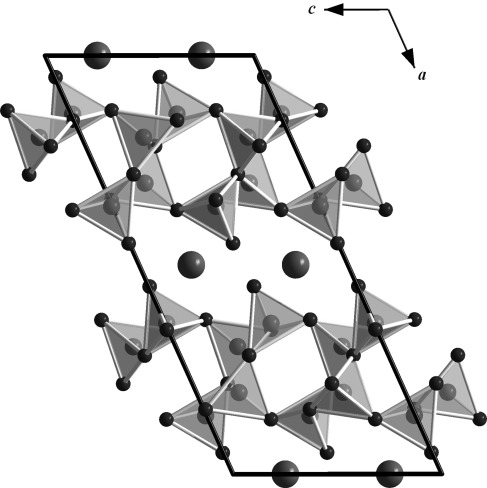
Crystal structure of the new actinide borates *An*B_4_O_8_ (*An=*Th, U; space group *C*2/*c*) along the *b* axis, which shows layers of linked [BO_4_^5−^] groups.

**Figure 3:**
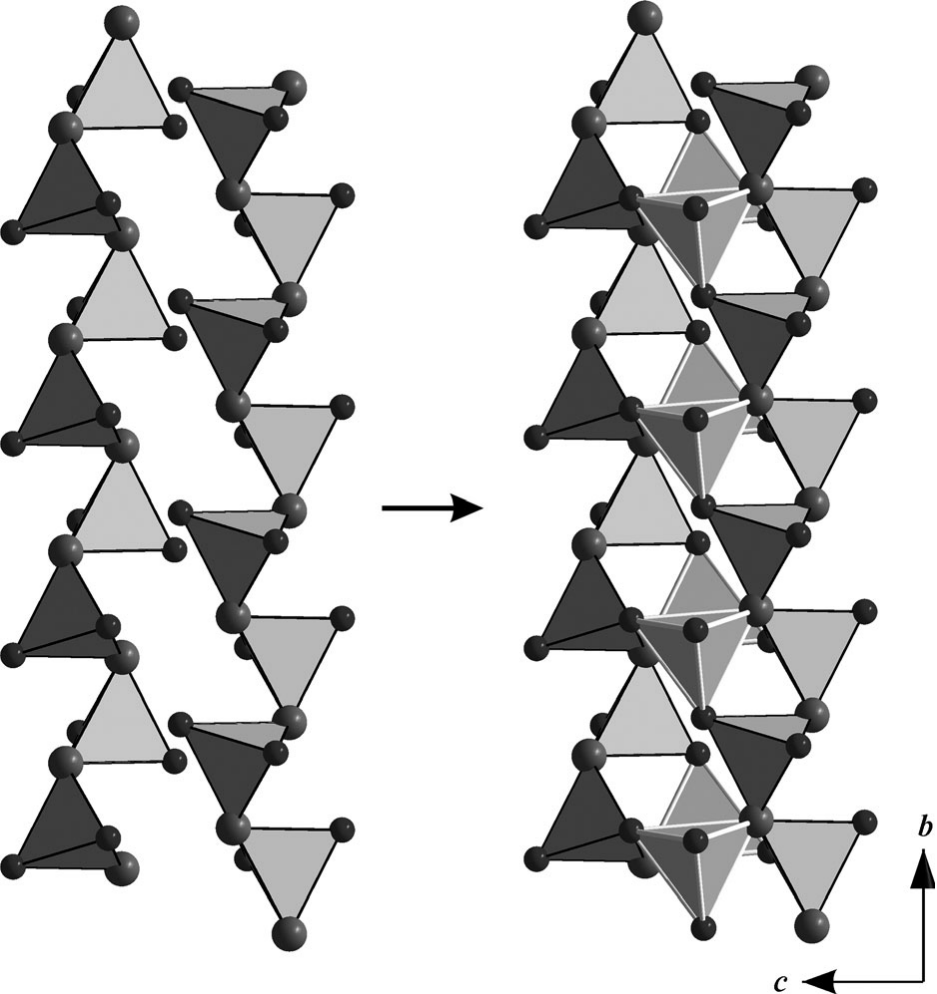
Crystal structure of *An*B_4_O_8_ (*An=*Th, U; space group *C*2/*c*). Left: Chains of corner-sharing [B2O_4_]^5−^ tetrahedra along the *b* axis, which are linked by the threefold coordinated O3 atoms (large spheres). Right: [B1O_4_]^5−^ tetrahedra (light-colored edges) link together two borate chains through oxygen atoms O2, O3, and O4.

**Figure 4:**
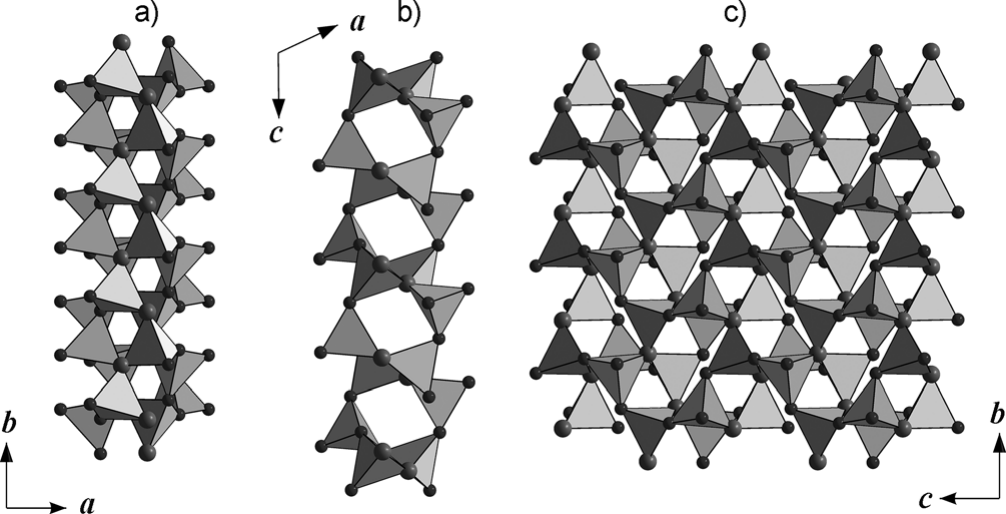
Crystal structure of *An*B_4_O_8_ (*An=*Th, U; space group *C*2/*c*), which shows the borate layers in the *bc* plane.

**Figure 5:**
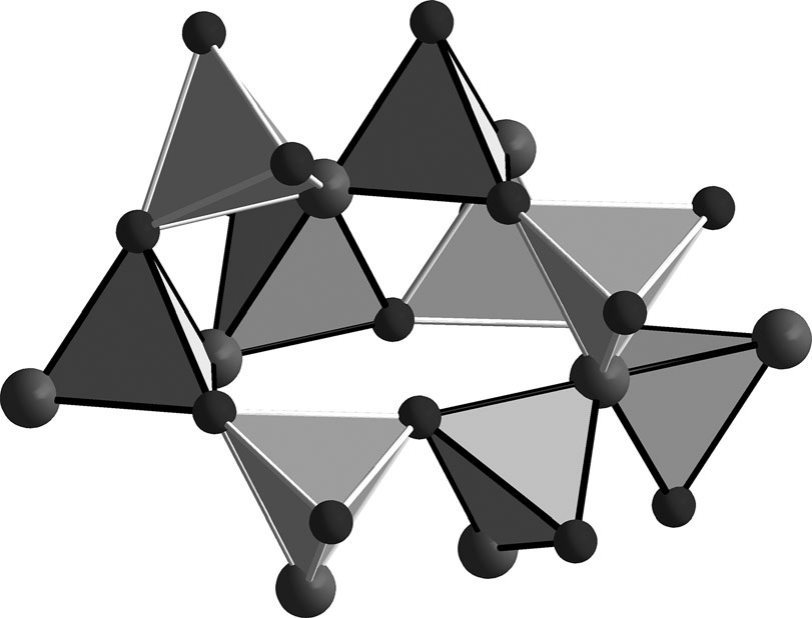
Unit of nine BO_4_ groups, which contains a central “dreier ring”, a “vierer ring”, and different “sechser rings”. [B1O_4_]^5−^ tetrahedra are denoted by light-colored edges and [B2O_4_]^5−^ tetrahedra are denoted by black edges. The threefold-coordinated oxygen atoms O3^3^ are denoted as large spheres.

**Figure 6:**
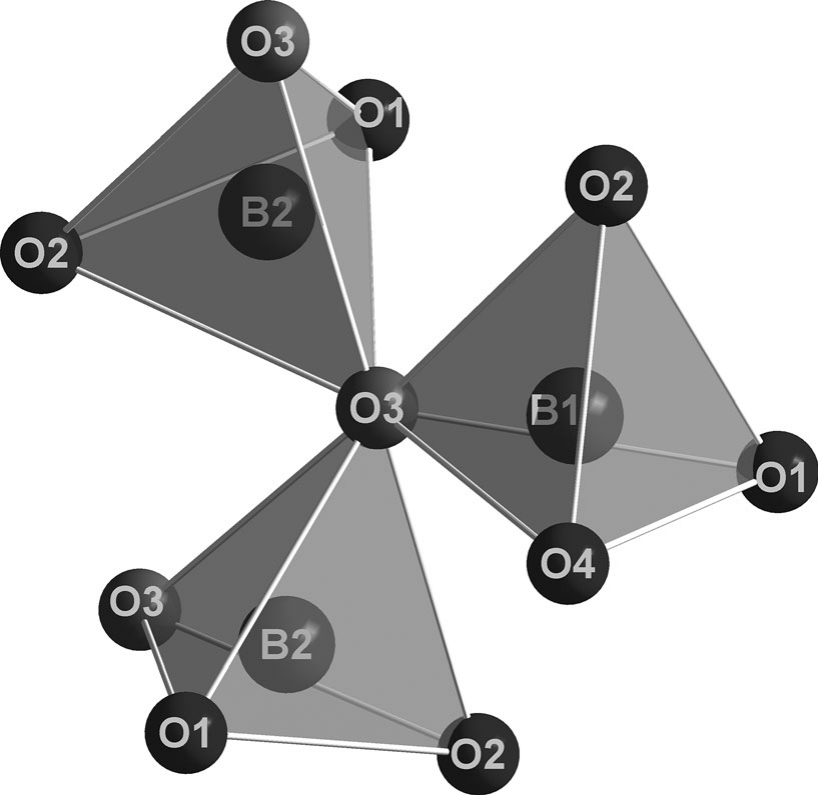
Threefold-coordinated oxygen atom (central sphere) in the crystal structure of *An*B_4_O_8_ (*An=*Th, U; space group *C*2/*c*).

**Figure 7:**
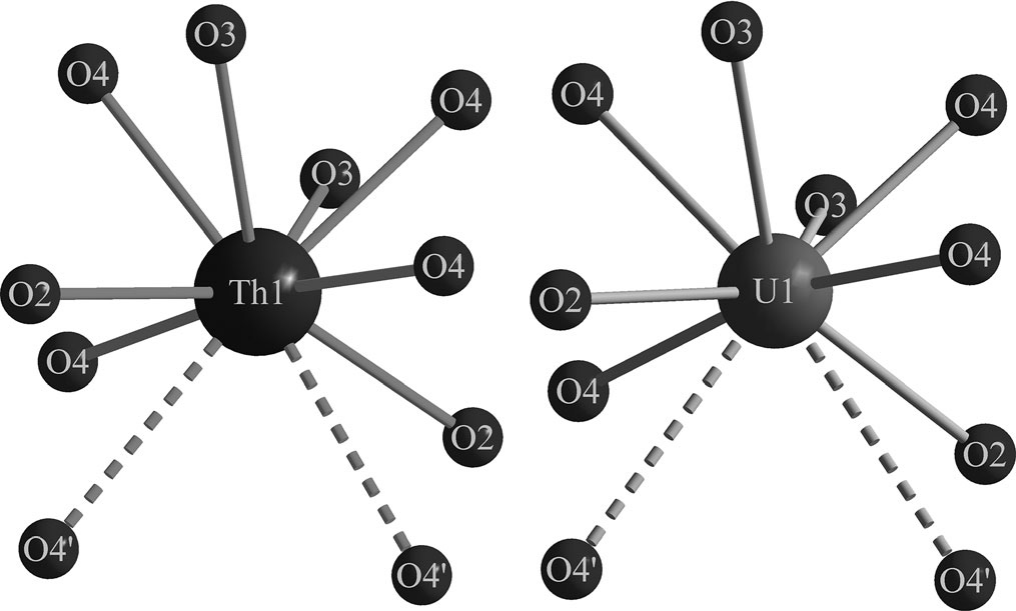
Coordination spheres of the actinide ions in *An*B_4_O_8_ (*An=*Th, U; space group *C*2/*c*); both cations are surrounded by ten oxygen anions, which possess 8+2-fold coordination.

**Crystal structures**: The new isotypic actinide borates ThB_4_O_8_ and UB_4_O_8_ crystallize in the monoclinic space group *C*2/*c*, with four formula units per unit cell. Lattice parameters for ThB_4_O_8_: *a*=1611.3(3), *b*=419.86(8), *c*=730.6(2) pm; *β*=114.70(3)°; *V*=449.0(2) Å^3^. Lattice parameters for UB_4_O_8_: *a*=1589.7(3), *b*=422.14(8), *c*=723.4(2) pm; *β*=114.13(3)°; *V*=443.1(2) Å^3^. Figure 2 shows the crystal structure of *An*B_4_O_8_ *(An=*Th, U) along the *b* axis, which is comprised of layers of corner-sharing [BO_4_]^5−^ tetrahedra that are separated by layers of actinide cations. As in most high-pressure borates, such as *RE*_4_B_6_O_15_ (*RE*=Dy, Ho^2^, ^17^), *α*-*RE*_2_B_4_O_9_ (*RE*=Sm–Tb, Ho^18^–^21^), and the rare-earth meta-borates *δ*-*RE*(BO_2_)_3_ (*RE*=Ce, La^22^, ^23^), this structure is exclusively built up from tetrahedral borate groups. Figure 3 shows the composition of the borate layers. A closer look at the layers exhibits infinite chains along the *b* axis, which consist of [B2O_4_]^5−^ tetrahedra that are connected through one common oxygen atom, O3 (Figure 3, large spheres). These chains of [B2O_4_]^5−^ tetrahedra and antiparallel-orientated chains alternate along the *c* axis and are linked together through the common oxygen atoms of the [B1O_4_]^5−^ and [B2O_4_]^5−^ tetrahedra (O1, O2, and O3). Figure 4 shows the layers along the *a*, *b*, and *c* directions. The BO_4_ groups form a central “dreier ring”, a “vierer ring“, and different “sechser rings”.^24^ The corners of the “dreier rings” are formed from two O3 atoms and one O2 atom that are located along the *b* axis. The “vierer rings” are composed of two [B1O_4_]^5−^ groups and two [B2O_4_]^5−^ groups that are linked together through the O2 and O3 atoms; these “vierer rings” form empty channels along the *b* axis, as shown in Figure 4. These rings can be represented by a unit that is comprised of five [B2O_4_]^5−^ groups and four [B1O_4_]^5−^ groups, as shown in Figure 5. The crystal structure of *An*B_4_O_8_ *(An=*Th, U) contains four crystallographically distinguishable oxygen atoms: Oxygen atoms O1 and O2 are twofold-coordinated by boron atoms. Oxygen atom O4 is a terminal oxygen atom that is orientated towards the cation layer; a view along the *c* axis shows the terminal O4 oxygen atoms of the borate layers. The O3 oxygen atom is exceptional, in that it is a threefold-coordinated oxygen atom. Figure 6 shows the [O3^[3]^(BO_3_)_3_]^11−^ unit, which is comprised of two [B2O_4_]^5−^ tetrahedra and one [B1O_4_]^5−^ tetrahedron. The B-O3^3^-B angles sum up to 360°, as expected from the trigonal-planar geometry. The two crystallographically distinguishable boron atoms in the actinide borates ThB_4_O_8_ and UB_4_O_8_ are tetrahedrally coordinated by four oxygen atoms. The mean values of the boron–oxygen distances inside the tetrahedra vary between 144.3(8) (B1–O2) and 152.1(6) pm (B2–O1), with a mean value of 147.2 pm for ThB_4_O_8_, and between 144.0(6) (B1–O3) and 152.1(7) pm (B2–O1), with a mean value of 147.2 pm for UB_4_O_8_. These values agree well with the known average value for the B–O distance in [BO_4_]^5−^ groups (147.6 pm).^25^–^27^ The oxygen-boron-oxygen angles in the tetrahedral [BO_4_]^5−^ groups in *An*B_4_O_8_ *(An=*Th, U) are listed in Table 5 and correspond well with the expected angles of tetrahedrally coordinated groups. Figure 7 shows the coordination sphere of the actinide cations in *An*B_4_O_8_ *(An=*Th, U). Ten oxygen atoms coordinate to both the thorium and uranium cations. Owing to the two longest actinide–oxygen distances (Th1–O4′′=285.9(5) pm (×2), U1–O4′′=300.2(4) pm (×2)) and the large difference between the third-longest *An*–O distances (Th1–O3=259.7(5) pm, U1–O3=252.2(4) pm), the description as an 8+2 coordination mode for the Th1 and U1 atoms is reasonable. These coordination spheres result in thorium–oxygen distances of between 240.3(5) and 285.9(5) pm, with a mean value of 255.9 pm for Th1 in ThB_4_O_8_, and between 231.4(4) and 300.2(4) pm, with a mean value of 253.7 pm for the uranium cations in UB_4_O_8_. A closer look at the lattice parameters (*a*, *b*, and *c; β*) reveals a decrease in the *a* (−1.3 %), *c* (−1.0 %), and *β* parameters (−0.5 %), in contrast to a slight increase in the *b* parameter (+0.5 %) in the structures of UB_4_O_8_ and ThB_4_O_8_. This divergence emerges from the higher ionic radius of Th^4+^. As expected, the *An*–O (*An*=U, Th) distances in ThB_4_O_8_ are larger, because of the larger ionic radius of Th^4+^. A comparison of the tetrahedral borate groups shows no greater deviances in the bond lengths and angles. In addition, the bond-valence sums for all atoms of ThB_4_O_8_ and UB_4_O_8_ were calculated by using the bond-length/bond-strength (ΣV)^28^, ^29^ and CHARDI concepts (charge distribution in solids, ΣQ).^30^ The results of these calculations are listed in Table 6. All of the calculated values correspond well with the expected values of the formal ionic charges.

**Table 6:** Charge distribution in *An*B_4_O_8_ (*An=*Th, U; space group *C*2/*c*), as calculated by using the bond-length/bond-strength (∑V)^28^, ^29^ and Chardi concepts (∑Q)^30^

	Th1	B1	B2	O1	O2	O3	O4
∑V	+3.79	+3.05	+3.07	−2.09	−2.00	−1.92	−2.00
∑Q	+3.95	+3.03	+3.00	−1.94	−2.06	−1.96	−2.04
	U1	B1	B2	O1	O2	O3	O4
∑V	+3.75	+3.01	+3.11	−2.05	−1.95	−1.99	−1.92
∑Q	+3.92	+3.05	+2.99	−1.89	−2.01	−2.06	−2.04

Owing to the fact that the structure type of *An*B_4_O_8_ *(An=*Th, U) is exclusively built up from BO_4_ tetrahedra, one could imagine a structural relationship with the structures of well-known uranium and thorium silicates. However, both of the ThB_4_O_8_ and UB_4_O_8_ structures exhibit threefold-coordinated oxygen atoms, a structural motif that is unknown in the chemistry of silicates. Therefore, there is no visible direct structural relationship.

MAPLE values (madelung part of lattice energy)^31^–^33^ were calculated for comparison with the MAPLE values as obtained from the summation of the binary actinide oxides, ThO_2_^34^ and UO_2_,^35^ and with that of the high-pressure modification B_2_O_3_-II.^36^ Values of 55 317 and 55 569 kJ mol^−1^ were obtained for ThB_4_O_8_ and UB_4_O_8_, respectively, in comparison with 55 421 kJ mol^−1^ (deviation=0.2 %) and 55 721 kJ mol^−1^ deviation=0.3 %) for the corresponding binary oxides (ThO_2_ (11 544 kJ mol^−1^)+2 B_2_O_3_-II (2×21 938 kJ mol^−1^); UO_2_ (11 544 kJ mol^−1^)+2×B_2_O_3_-II (2×21 938 kJ mol^−1^)).

**Figure 8:**
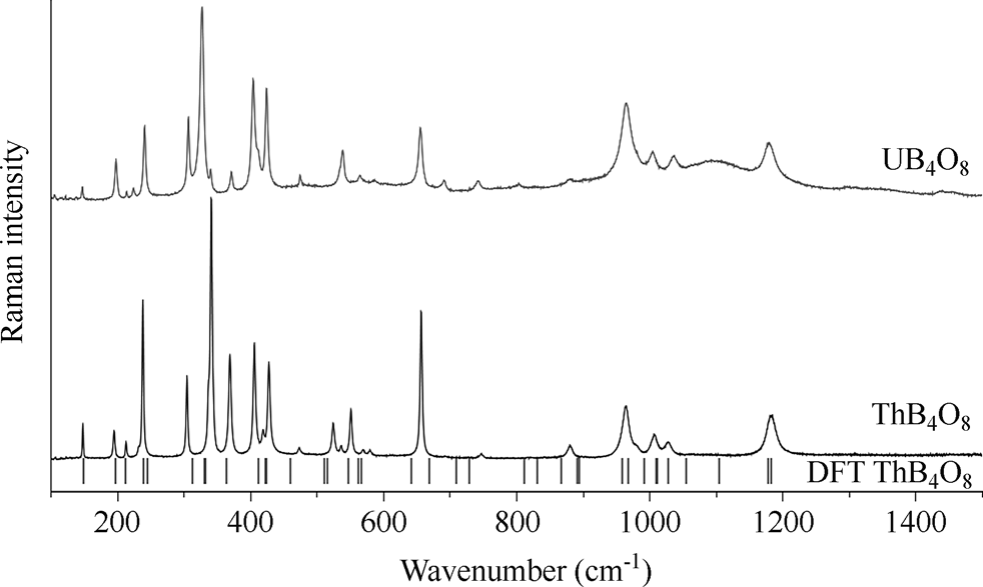
Raman spectra of single crystals of UB_4_O_8_ (top) and ThB_4_O_8_ (bottom) and theoretical bands of ThB_4_O_8_ (lines) within the range 100–1500 cm^−1^.

**Vibrational spectroscopy**: Figure 8 shows the Raman spectra of single crystals of the actinide borates ThB_4_O_8_ and UB_4_O_8_ within the range 100–1500 cm^−1^. No OH or water bands could be detected within the range 3000–3600 cm^−1^. Bands at about 900 cm^−1^ in borate compounds are usually assigned to the stretching modes of the [BO_4_]^5−^ groups. However, trigonal [BO_3_]^3−^ groups are expected at wavenumbers above 1150 cm^−1^.^[37–40]^ No bands are observed above 1200 cm^−1^, as expected from the crystal structure due to the absence of trigonal [BO_3_]^3−^ groups. Bands at smaller wavenumbers than 500 cm^−1^ can be assigned to *An*–O (*An*=Th, U) bonds, to lower-wavenumber-shifted bending and stretching modes of tetrahedral [BO_4_]^5−^ groups, and to lattice vibrations. The large variation in B–O distances and in the linkage of the tetrahedral [BO_4_]^5−^ groups led to various experimentally observed modes.

FTIR-ATR measurements of the products (mixture of the actinide borate, unreacted actinide oxide, and a still-unknown phase) were performed to exclude water or hydrated borates. The spectra showed no bands within the region 3000–4000 cm^−1^.

**Quantum-mechanical calculations of the harmonic vibrational frequencies**: To validate the quality of the basis sets and the functional, a geometry optimization of ThB_4_O_8_ was performed. Starting from the single-crystal structure, the geometry optimization yielded deviations in the lattice parameters and the atomic positions of less than 1 %. The calculations of the harmonic vibrational frequencies were performed on the optimized geometry. The calculated Raman bands fit quite well with the experimental spectrum of single crystals of ThB_4_O_8_. The low deviations were obtained from the approximations in the DFT method and the calculations on just one unit cell. Calculations of larger systems (super cells of ThB_4_O_8_) were not possible. Moreover, the calculation did not consider the temperature (297 K for the experiment) and the addition of two Gaussian peaks in the experimental spectrum led to a shift of the maxima. Table 7 lists the modes above 600 cm^−1^. These bands can be assigned to boron–oxygen bending or stretching modes. However, in the assignment, the highly condensed boron–oxygen layers must be considered. An exclusive stretching or bending motion inside a tetrahedral [BO_4_]^5−^ group was not possible. Each stretching or bending motion induced motions of neighboring atoms. As expected, the calculation yielded no vibrational modes above 1200 cm^−1^. The evaluation of the theoretical modes exclusively showed the impossibility of the assignment of one band to a particular stretching or bending mode inside the [BO_4_]^5−^ group. For example, the two modes at 1179 and 1182 cm^−1^ derive their origin from a various number of vibrational modes in the boron–oxygen layer.

**Table 7:** Comparison and assignment of selected theoretical and experimental boron–oxygen bands in the Raman spectra of ThB_4_O_8_^[a]^

Theoretical band [cm^−1^]	Experimental band [cm^−1^]	Assignment
1179, 1182	1183	b(B1-O3-B2), b(O1-B1-O2), b(O2-B2-O1), b(O2-B2-O3), s(B2–O4)
1056	1077	s(B2–O2)
1029	1028	s(B1–O2), b(O3-B2-O2), b(O4-B2-O2)
1010, 1013	1008	s(O3-B1-O2), s(B2–O4), b(O2-B2-O4), b(O3-B2-O4)
992	981	s(O2-B2-O4)
959, 969	964	s(O3-B1-O2), s(O4-B2-O3), s(B2–O3)
868, 892	880	s(B1–O1), b(O1-B2-O2)
643, 669	657	b(O2-B1-O3), b(O2-B2-O3), b(O4-B2-O3)

[a] s=Stretching mode, b=bending mode; pairs of bonded atoms with a large relative motion between them are given in parentheses.

## Conclusion

Herein, we have described the high-pressure, high-temperature syntheses, single-crystal structural determinations, spectroscopic investigations, and theoretical calculations of the new actinide borates ThB_4_O_8_ and UB_4_O_8_. The crystal structures are constructed from layers of linked BO_4_ tetrahedra. These layers contain threefold-coordinated oxygen atoms. The actinide cations are located between the boron–oxygen layers. In the future, we will attempt the synthesis of isotypic compounds with other cations in the oxidation state 4+ that have similar ionic radii, such as Ce^4+^, by using the multianvil high-pressure technique. Furthermore, this research into actinide borates will be a good starting point for synthesizing the first actinide fluoride borate, in analogy to our work in the field of rare-earth fluoride borates.

## Experimental Section

Caution: Working with UO_3_ and ThO_2_ requires appropriate precautions for the handling of radioactive and toxic substances.

**Synthesis**: The syntheses of *An*B_4_O_8_ *(An=*Th, U) took place under high-pressure, high-temperature conditions. The synthesis of ThB_4_O_8_ was carried out at 5.5 GPa and 1100 °C, whilst the isotypic compound UB_4_O_8_ was synthesized at 10.5 GPa and 1100 °C. Depending on the actinide borate, stoichiometric mixtures of ThO_2_ (synthesized by the decomposition of Th(NO_3_)_4_**⋅**4 H_2_O at 750 °C) or UO_3_ (synthesized by the pyrolysis of UO_2_(NO_3_)_2_**⋅**6 H_2_O at 300 °C) and B_2_O_3_ (Strem Chemicals, +99.9 %) in a 1:2 molar ratio were finely ground together, added into a platinum capsule, and placed in a boron-nitride crucible (Henze BNP GmbH, HeBoSint S100, Kempten, Germany). Then, the crucibles were placed into the center of an 18/11 assembly (for the thorium borate) or into the center of a 14/8 assembly (for the uranium borate). All of the synthetic steps were performed inside a glove box. The assemblies were compressed by using eight tungsten-carbide cubes (TSM-10 Ceratizit, Reutte, Austria). To apply the pressure, a 1000 ton multianvil press with a Walker-type module (both devices were purchased from Voggenreiter, Mainleus, Germany) was used. The assembly and its preparation are described in refs. 41–45. For the synthesis of ThB_4_O_8_, the 18/11 assembly was compressed up to 5.5 GPa over 160 min, then heated at 1100 °C (in a cylindrical graphite furnace) over 10 min, kept at that temperature for 10 min, and cooled to 450 °C over 25 min at constant pressure. UB_4_O_8_ was synthesized by compressing the 14/8 assembly up to 10.5 GPa over 280 min, then heated at 1100 °C (in a cylindrical graphite furnace) over 10 min, kept at that temperature for 10 min, and cooled to 450 °C over 25 min at constant pressure. After natural cooling to RT by switching off the heating, decompression periods of 8 and 14 h were required. The recovered octahedral pressure medium (MgO, Ceramic Substrates & Components Ltd., Newport, Isle of Wight, UK) was broken apart and the samples were carefully separated from the surrounding graphite and boron nitride. Whilst ThB_4_O_8_ was obtained as colorless crystals, UB_4_O_8_ was obtained as green, air- and water-resistant crystals in a black matrix.

**Crystal-structure analysis**: The powder X-ray diffraction pattern of *An*B_4_O_8_ *(An=*Th, U) were obtained in transmission geometry from flat samples of the reaction product on a STOE STADI P powder diffractometer with Mo_K*α*1_ radiation (Ge monochromator, *λ*=70.93 pm). Figure 1 shows the experimental powder X-ray diffraction patterns of ThB_4_O_8_ and UB_4_O_8_, which matched well with the theoretical patterns that were simulated from the single-crystal data. The respective diffraction patterns showed reflections of ThB_4_O_8_ or UB_4_O_8_, unreacted ThO_2_ or UO_2.12_ (denoted with lines in Figure 1), and, in both cases, a still-unknown phase (denoted with circles in Figure 1). Small single crystals of ThB_4_O_8_ and UB_4_O_8_ were isolated by mechanical fragmentation. The single-crystal intensity data were collected at RT on a Nonius Kappa-CCD diffractometer with graphite-monochromated Mo_Kα_ radiation (*λ*=71.073 pm). A semiempirical absorption correction based on equivalent and redundant intensities (Scalepack^46^) was applied to the intensity data. All of the relevant details of the data collection and evaluation are listed in Table 1 for both compounds. The structure solution and parameter refinement (full-matrix least-squares against *F*^2^) were performed by using the SHELX-97 software suite.^47^, ^48^ According to the systematic extinctions, the monoclinic space group *C*2/*c* was derived in both cases. All of the atoms were refined with anisotropic displacement parameters and the final difference Fourier syntheses did not reveal any significant peaks in both refinements. Tables 2–3, 4, 5, 6 list the positional parameters, anisotropic displacement parameters, interatomic distances, and angles in these structures.

CSD-426310 (ThB_4_O_8_) and CSD-426311 (UB_4_O_8_) contain the supplementary crystallographic data for this paper. These data can be obtained from the Fachinformationszentrum Karlsruhe via http://www.fiz-informationsdienste.de/en/DB/icsd/depot_anforderung.html.

**Vibrational spectra**: Confocal Raman spectra of single crystals of *An*B_4_O_8_ *(An=*Th, U) within the range 50–4000 cm^−1^ were recorded on a Horiba Jobin Yvon Labram-HR 800 Raman microspectrometer. The samples were excited by using the 532 nm emission line of a frequency-doubled 100 mW Nd:YAG laser and by using the 633 nm emission line of a 17 mW HeNe laser with an Olympus ×50 objective lens. The diameter of the laser spot on the surface was approximately 1 μm. The scattered light was dispersed by using an optical grating with 1800 lines mm^−1^ and collected by using a 1024×256 open-electrode CCD detector. The spectroscopic resolution, as determined by measuring the Rayleigh line, was less than 2 cm^−1^. The spectra were recorded unpolarized. The accuracy of the Raman line shifts, as calibrated by regularly measuring the Rayleigh line, was on the order of 0.5 cm^−1^. Background and Raman bands were fitted by using the built-in spectrometer software LabSpec to a second-order polynomial and convoluted Gaussian–Lorentzian functions, respectively.

The FTIR-ATR (Attenuated Total Reflection) spectra of powdered samples were measured on a Bruker Alpha-P spectrometer with a diamond ATR-crystal (2×2 mm) that was equipped with a DTGS detector within the spectroscopic range 400–4000 cm^−1^ (resolution: 4 cm^−1^). 24 scans of the sample were acquired. A correction for atmospheric conditions was performed by using OPUS 7.0 software.

**DFT calculations**: In addition to the experimentally recorded IR and Raman spectra of ThB_4_O_8_, quantum-chemical computations of harmonic vibrational frequencies were performed by using the Crystal 09 program.^49^–^51^ An important step in any quantum-mechanical calculation is the choice of an adequate basis set and a compromise must often be found between balancing computational effort and the accuracy of the results. To decrease the computational effort, a basis set with an effective core potential (ECP) for thorium was chosen. A suitable basis set for the actinide atom was identified based on geometry optimizations of ThB_4_O_8_. All-electron basis sets were employed for boron^52^ and oxygen atoms.^53^ Out of the results on the geometry optimization of ThB_4_O_8_, the well-tested ECP78MWB GUESS^54^ basis set was chosen for the thorium atom. All of the calculations were performed by using the PBESOL functional^55^ for the correlation and exchange functionals and the SCF convergence for the energy was set at ×10^−12^ E_h_. The overall computation time for the calculations of the harmonic vibrational frequencies of ThB_4_O_8_ took 168 h on a cluster with 12 Intel Xeon CPU X5670 2.93 GHz processors.
